# Host country responses to non-communicable diseases amongst Syrian refugees: a review

**DOI:** 10.1186/s13031-019-0192-2

**Published:** 2019-03-22

**Authors:** Chaza Akik, Hala Ghattas, Sandra Mesmar, Miriam Rabkin, Wafaa M. El-Sadr, Fouad M. Fouad

**Affiliations:** 1 Center for Research on Population and Health, Faculty of Health Sciences, American University of Beirut, Beirut, Lebanon; 2 ICAP, Columbia University Mailman School of Public Health, New York, USA; 3 Department of Epidemiology and Population Health, Faculty of Health Sciences, American University of Beirut, Beirut, Lebanon

**Keywords:** Syrian refugees, Non-communicable diseases, Health systems, Lebanon, Jordan, Turkey

## Abstract

**Background:**

Since the beginning of the Syrian conflict in 2011, Jordan, Lebanon and Turkey have hosted large refugee populations, with a high pre-conflict burden of non-communicable diseases (NCDs).

**Objectives:**

We aimed to describe the ways in which these three host country health systems have provided NCD services to Syrian refugees over time, and to highlight the successes and challenges they encountered.

**Methods:**

We conducted a descriptive review of the academic and grey literature, published between March 2011 and March 2017, using PubMed and Google searches complemented with documents provided by relevant stakeholders.

**Results:**

Forty-one articles and reports met our search criteria. Despite the scarcity of systematic population-level data, these documents highlight the high burden of reported NCDs among Syrian refugees, especially amongst older adults. The three host countries utilized different approaches to the design, delivery and financing of NCD services for these refugees. In Jordan and Lebanon, Ministries of Health and the United Nations High Commissioner for Refugees (UNHCR) coordinate a diverse group of health care providers to deliver health services to Syrian refugees at a subsidized cost. In Turkey, however, services are provided solely by the Disaster and Emergency Management Presidency (AFAD), a Turkish governmental agency, with no cost to patients for primary or secondary care. Access to NCD services varied both within and between countries, with no data available from Turkey. The cost of NCD treatment is the primary barrier to accessing healthcare, with high out-of-pocket payments required for medications and secondary and tertiary care services, despite the availability of free or subsidized primary health services. Financial impediments led refugees to adopt coping strategies, including returning to Syria to seek treatment, with associated frequent treatment interruptions. These gaps were compounded by health system related barriers such as complex referral systems, lack of effective guidance on navigating the health system, limited health facility capacity and suboptimal NCD health education.

**Conclusion:**

As funding shortages for refugee services continue, innovative service delivery models are needed to create responsive and sustainable solutions to the NCD burden among refugees in host countries.

**Electronic supplementary material:**

The online version of this article (10.1186/s13031-019-0192-2) contains supplementary material, which is available to authorized users.

## Introduction

Forcible displacement is increasing globally, affecting an estimated 68.5 million people in 2017 [1]. In Syria, armed conflict has caused massive internal and external displacement, including an exodus of over 5 million people into its neighboring countries [2]. As a result, one in six people in Lebanon is a refugee – the highest proportion of refugees to host population in the world. In Jordan, 1 of every 14 people is a refugee, whereas Turkey hosts 3.5 million people, the largest absolute number of refugees in the world [3].

The influx of Syrian refugees has placed significant pressures on existing services within hosting countries, particularly on health and education. The responses to this complex emergency has differed by host country; while Jordan and Turkey established refugee camp infrastructure, Lebanon did not, with refugees living either in host communities, or in informal tented settlements where access to essential services such as shelter, food, sanitation, and health care is not officially established. In all three countries, the majority of refugees live in host communities (79, 83, and 92% respectively) [4, 5].

Prior to the influx of refugees, Jordan had a strong public health care system with an extensive network of primary health care facilities [6]. Lebanon had a highly privatized health care system with a nascent network of primary health care clinics largely managed by NGOs [7]; and in Turkey, the Ministry of Health is the main actor; universities and the private sector are also providers of services [8].

Each of these health systems has been challenged to respond to the diverse health needs of Syrian refugees while maintaining services for their own citizens. In addition to the need for emergency and basic health services such as reproductive and maternal/child health, displaced Syrians have a disease burden profile consistent with that of middle-income countries, including the predominance of chronic non-communicable diseases (NCDs). Prior to the beginning of the conflict, the leading causes of morbidity and mortality in Syria were cardiovascular diseases and Type II diabetes mellitus with 77% of deaths attributed to NCDs [9, 10].

Although refugee health services are traditionally conceptualized as largely acute and/or episodic, the protracted nature of the crisis and the high prevalence of NCDs amongst Syrians required host country health responses to also design and deliver continuity care, including the prevention, detection and treatment of NCDs [11]*.* The diverse health systems and stakeholders in Jordan, Lebanon and Turkey, contributed to a greatly varied approach to the provision of these services, and to changes over time in the three countries. To date, these responses have not been systematically analyzed and compared to draw lessons for programs and policy.

We therefore conducted a descriptive review of the academic and grey literature, aiming to characterize the ways in which Jordan, Lebanon and Turkey provided NCD services to Syrian refugees over time, and to highlight the successes and challenges they encountered. We reviewed the current landscape in terms of: 1) NCD burden; 2) NCD service provision; 3) access to NCD services and medications; and 4) barriers and facilitators to accessing NCD care by Syrian refugees.

## Methods

### Search strategy

We used a three-pronged approach: retrieving academic research, reviewing reports published in the grey literature, and consulting with relevant stakeholders to obtain additional unpublished data. We searched PubMed for published articles on NCD burdens among Syrian refugees, risk factors for NCDs, access to and utilization of services, systems of care for refugees in camps and non-camp settings, capacity of workforce, quality and acceptability of care and financing for Syrian refugee populations’ health services. We restricted our search terms to: arthritis, asthma, cancer, cardiovascular diseases, respiratory diseases, coronary heart disease, diabetes, hypertension, hypertriglyceridemia, metabolic syndrome, osteoporosis, renal diseases, and stroke. These terms for NCD conditions and their common risk factors, as well as terms related to health care systems, services and access, and Syria/Syrian constituted the PubMed search strategy, both as MeSH and key terms (Additional file 1). No language restrictions were placed on the search. Peer-reviewed studies published between March 2011 and March 2017 were included, and opinion articles excluded. Google Scholar was searched for additional articles published in Turkish.

Three researchers fluent in Arabic, English and Turkish ran a complementary search by reviewing the grey literature separately for each of the three countries using Google for the same time period. Wider search terms were used to retrieve any NCD and included Syria, refugees and chronic diseases. We included annual reports, mid-year reports, one-time reports and nationally representative surveys addressing the outcomes listed above. We excluded weekly and quarterly programmatic reports, as well as journalistic articles.

We also contacted 48 stakeholders who provide services to Syrian refugees in the three countries by email, including staff of governmental, and local and international non-governmental organizations as well as academics to request additional reports relevant to the topic of interest.

For published articles to be included, they needed to fulfill quality criteria informed by the Preferred Reporting Items for Systematic Reviews and Meta-Analyses (PRISMA) guidelines [12], including clear eligibility criteria for study selection, description of information sources, data and variables; we excluded studies that did not report on methodology, and those that presented unclear or inconsistent numbers. For the grey literature, quality appraisal took into account whether the publication was data driven (based on original or secondary data) or not, and whether there was a specified study methodology.

### Data extraction and synthesis

Articles and reports retrieved were imported into Endnote X7 and duplicates were removed. Title, abstract and full-text screening were conducted to retrieve relevant studies. We used the open-source Open Data Kit (ODK) (https://ona.io/) to create the data entry protocol. The data extracted for each study included: document identification (title, authors/organization, type of literature, URL, year of publication, language), research design, reported chronic diseases and their prevalence, access to health services, reported risk factors for outcomes (e.g. diet, smoking, overweight/obesity), financing systems and systems of chronic disease care, and capacity and quality and acceptability of chronic disease care. A number of included articles were based on survey reports retrieved in the search; we report data from the original peer-reviewed articles rather than survey reports when duplicate information were presented. A thematic analysis was adopted for data synthesis.

## Results

**Fig. 1:**
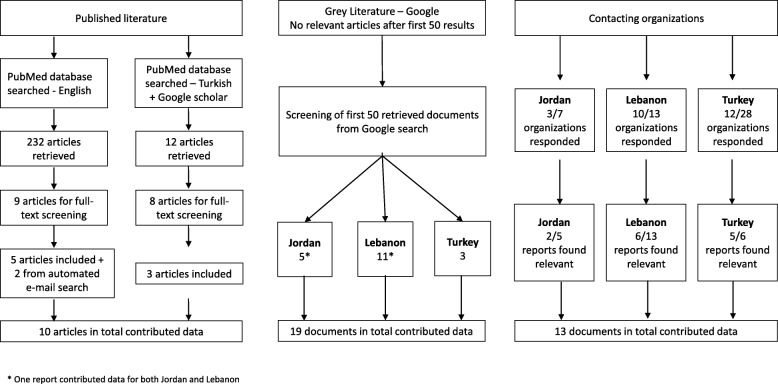
Screening flowchart for published and grey literature, and stakeholder-contributed documents

The review of published literature retrieved 244 articles, of which 10 contributed data to our analysis (Fig. 1). Nine out of 10 studies were cross-sectional using mixed methods (*n* = 2) or quantitative instruments; one was a retrospective analysis of clinical records; and one was a historical discourse analysis. Screening of the grey literature retrieved 19 relevant reports. Twenty-five of 48 stakeholders provided 24 documents, 13 of which contributed data. Figure 1 presents the specific numbers by country of interest. All data presented as part of this descriptive review cover the period March 2011–March 2017, with the latest policy changes highlighted in the discussion.

We first present the evidence on burden of NCDs among Syrian refugees, the health systems strategies in host countries and access to NCD services for these refugees; followed by the barriers to accessing NCD services. We found no information on models of NCD care, continuity or quality of care or adherence to care.

### Burden of non-communicable diseases among Syrian refugees

Although systematic population-level data are scarce, the available information indicates a high burden of reported NCDs among Syrian refugees living in neighboring host countries (Table 1), with the highest prevalence amongst older adults.

**Table 1:** Burden of NCDs among Syrian refugees in Jordan, Lebanon and Turkey

Country	Survey (Year)	Target population	Any NCD condition	Hypertension	Diabetes mellitus	Cardiovascular diseases	Chronic respiratory diseases	Lung disease	Arthritis	Osteoarthritis	Renal disease	Liver disease	Cancer
Nationally representative surveys													
Jordan	Syrian refugee health access survey in Jordan (2014) [13]	HH^a^	50.3	–	–	–	–	–	–	–	–	–	–
		Adult	–	10.7	6.1	4.1	2.9	–	7.1	–	–	–	–
Turkey	Non-communicable disease risk factors surveillance among Syrian refugees living in Turkey (2016) [14] Self-reported	Adult	–	10.2	5.1	–	–	–	–	–	–	–	–
	Measured	Adult		25.6									
Non-nationally representative community-based surveys													
Lebanon	Prevalence, care-seeking, and health service utilization for non-communicable diseases among Syrian refugees and host communities in Lebanon (2016) [15]	HH^a^	50.4	–	–	–	–	–	–	–	–	–	–
Turkey	Rapid Need Assessment of Gaziantep based Syrian refugees (2015) [19]	HH^a^	25	–	–	–	–	–	–		–	–	–
Turkey	Evaluation of health status and health service utilization of refugees living in Zeytinburnu-Istanbul [20]	HH^a^	73.9	25.2	8.1	12.6	19.8	–	–	–	7.2		2.7
Jordan	Health access and utilization survey among non-camp Syrian refugees (2014)^d^ [17]	Adult	39.8	39.5	25.8	17.7	–	7.9	–	–	3.6	1.3	3.4
Lebanon	Prevalence, care-seeking, and health service utilization for non-communicable diseases among Syrian refugees and host communities in Lebanon (2016) [15]	Adult	–	7.4	3.3	3.3	3.8	–	7.9	–	–	–	–
Lebanon	Health access and utilization survey among non-camp Syrian refugees (2014)^c^ [16]	Adult	14.6	25.4	15.9	18.4	–	10.7	–	–	6.6	2.1	1.8
Jordan	Hidden victims of the Syrian crisis: disabled, injured and older refugees (2014) [18]	All ages	19.6	–	–	–	–	–	–	–	–	–	–
Lebanon	Hidden victims of the Syrian crisis: disabled, injured and older refugees (2014) [18]	All ages	13	–	–	–	–	–	–	–	–	–	–
Lebanon	MSF Health assessment project: Bekaa Valley Follow-Up Assessment (2014) [40]	All ages	–	34	14	–		–	–	–	8	3	3
Turkey	Syrian refugees in Turkey. Field survey results (2013) [21]												
	In camps	All ages	7.3	–	–	–	–	–	–	–	–	–	–
	Outside camps	All ages	7.6	–	–	–	–	–	–	–	–	–	–
Turkey	Health status, access to health and barriers for access to health of Syrian refugees in a district in Izmir^e^ (2016) [22]	All ages	–	3.8	2.9	–	1.6	–	–	0.4	0.2	–	0.5
Turkey	Syrian refugees needs analysis survey (2013) [23]	All ages	13.3										
Facility-based surveys													
Lebanon	Health status and health needs of older refugees from Syria in Lebanon (2015) [25]	> 60	–	53	38	28	11^b^	–	31	–	6	–	–
Jordan	Meeting the health needs of Syrian refugees in Jordan: a novel model for non-communicable disease care in a refugee setting (2015) [24]	All ages	–	53	51	–	1	–	–	–	–	–	–

^a^HH: Household

^b^ Asthma 18%

^c^Coronary heart diseases prevalence at 23.4%

^d^ Ischemic heart diseases prevalence of 2.6%

^e^ Ischemic heart diseases prevalence of 2.5%, hypercholesterolemia prevalence of 22% and neurologic disorders (including stroke) of 5%

### Nationally representative surveys

In Jordan, a 2014 survey of 1550 households found that half of the refugee households interviewed (50.3%) proxy-reported at least one member living with at least one of the five NCDs assessed [13]. Amongst adults, the most common conditions were hypertension, and arthritis, followed by diabetes mellitus, cardiovascular disease and chronic respiratory disease (Table 1). In Turkey, a 2015 WHO STEPwise approach to Surveillance (STEPS) survey of Syrian refugees living in and outside camps found that 5.1% of adults (18–69 years) self-reported being diabetic; and while only 10.2% self-reported being hypertensive, 25.6% were diagnosed as hypertensive (measured SBP ≥ 140 and/or DBP ≥ 90 mmHg or currently on medication for raised blood pressure) [14]. No data from nationally representative surveys were available for Lebanon during the search time period.

### Non-nationally representative community-based surveys^1^

In Lebanon, a 2014 health access survey assessed 1376 refugee households within host communities and in informal tented settlements in all governorates except for the South and several areas of the Bekaa region of the country [15]. Half of the households reported having at least one member living with at least one NCD (50.4%). Surveys of non-camp refugees in Jordan (*n* = 491 households) and Lebanon (*n* = 566 households in host communities or informal tented settlements) showed that 39.8 and 14.6% of adults, respectively, self-reported at least one NCD with prevalence reaching 53.9 and 46.6% among older adults (over 60 years of age) in Jordan and Lebanon, respectively [16, 17]. Similarly, in a survey among 3202 Syrian refugees in Jordan and Lebanon, the prevalence of NCDs was self-reported at 19.6 and 13% respectively, with prevalence reaching 54% among older adults (over 60 years of age) [18]. Amongst adults, reported hypertension and arthritis were most common, followed by chronic respiratory disease, diabetes mellitus and cardiovascular diseases (Table 1).

In Turkey, a survey among Syrian refugees in Gaziantep - a city near the Syrian border - reported that 25% of households included at least one member living with an NCD, whereas in another study of an Istanbul neighborhood, this number reached 74% of households. This wide range is likely due to the differences in the populations surveyed as well as methodological differences in the definition of NCDs included [19, 20]. Hypertension, chronic respiratory disease and cardiovascular disease were the most common (Table 1). A field survey in Turkey found a similar proxy-reported prevalence of NCD among refugees in camps (7.3%) and for those living outside camps (7.6%) [21]; similarly the burden of specific NCDs among Syrian refugees of any age in Izmir was self-reported to be low [22]. A needs assessment of Syrian women refugees in seven Turkish provinces reported a 13.3% prevalence of NCDs [23].

### Facility-based surveys

Facility-based surveys in Jordan and Lebanon reported high burden of NCDs. Among adult patients attending a Médecins Sans Frontières (MSF) clinic in Jordan (*n* = 778), 53% were hypertensive and 51% were diabetic [24]. Among the older refugee population attending Caritas Lebanon’s Migrant Center (*n* = 167), 53% self-reported being diagnosed with hypertension and 38% with diabetes mellitus [25] (Table 1).

### Health systems strategies in Jordan, Lebanon and Turkey

The three host countries have utilized different approaches to the design, delivery and financing of NCD services for Syrian refugees (Table 2). In Jordan and Lebanon, Ministries of Health and the United Nations High Commissioner for Refugees (UNHCR) coordinate a diverse group of health care providers to deliver health care to Syrian refugees, whereas in Turkey, services are provided solely by the Turkish government, led by the Disaster and Emergency Management Presidency (AFAD).

**Table 2:** Healthcare provision, referral policies, and financing schema for Syrian refugees in Lebanon, Jordan, and Turkey

	Jordan	Lebanon	Turkey
Health Care Provision	The Ministry of Health, UNHCR and partner non-governmental organizations (NGOs) such as Jordan Health Aid Society (JHAS). [56]. The Ministry of Health provides full access to health services for Syrian refugees outside camps. For camp refugees, primary and secondary healthcare is delivered inside camp through a host of NGO clinics, UNHCR and national organizations.	Around 100 primary healthcare centers (PHCs), that are under the umbrella of UNHCR, NGOs, and the Ministry of Social Affairs and the Ministry of Public Health. [39] MoPH and YMCA are responsible for distributing medication and laboratory supplies [26].	The Disaster and Emergency Management Presidency of Turkey (AFAD). Voluntary health centers run by non-governemntal organizations with special permission from the Ministry of Health also provide primary health care [29]. Community health centers provide primary health care services, while field hospitals and polyclinics provide secondary health services [28]. Refugee health centers work in alliance with community health centers to provide primary care health services to Syrian refugees [30].
Access to Healthcare	Refugees registered with UNHCR need to obtain a Ministry of Interior (MOI) service card to access primary, secondary and some tertiary healthcare at Ministry of Health (MOH) facilities [47].	Refugees registered with UNHCR obtain a registration card to access healthcare services at PHCs*.*	Refugees registered under the Temporary Protection Regime have the right to Social Security, subsidized by AFAD [29, 57]. Syrian refugees who reside in and out of the camps have free access to primary and secondary health care facilities [30, 57].
Financing Scheme			
Coverage of Costs	Access to primary and secondary care was free of charge for UNHCR registered refugees, until late 2014. Costs were not covered for medicines or private facilities. Due to a governmental policy change, refugees with a MOI service card are required to pay the same highly subsidized rate for care at PHC facilities as uninsured Jordanians at any healthcare facility attended [33, 47].	UNHCR Scheme: - Refugees pay a subsidized fee of USD 2–3.33 per consultation at PHC centres. -For refugees that are < 5 and > 60 years old, disabled people, pregnant and lactating women, 85% of diagnostic costs are covered. Other refugees pay 100% of these costs required for referral to hospital treatment or medicines [26]. -Those with chronic conditions pay USD 0.67 as handling fee for medication. Medications are free of charge through YMCA [42]. Other schemes: Privately funded PHCs may be providing free primary health care to registered and unregistered refugees, in accordance with their own guidelines [26].	Access to healthcare through social security is free in the geographic area in which the refugee has registered his/her social security [29]*.*
Unregistered refugees	Refugees without the MOI service card pay a “foreigner rate” which is up to 60% higher than the rate of uninsured Jordanians [33, 47].		Unregistered refugees pay to access services, like Turkish citizens without Social Insurance, except in cases of emergency, preventive health services and communicable diseases [28, 29].
Referral System Policy	Cases requiring secondary or tertiary care are referred to a neighboring hospital. Referral from PHCs to government hospitals are possible only if the refugee is able to pay the remaining rate of the subsidized cost [32]. UNHCR works with JHAS clinics to cover the cost of treatment for patients through JHAS clinics if the patient is unable to pay for the treatment at PHCs and governmental hospitals and is deemed vulnerable. Patients are referred to private affiliated hospitals. UNHCR’s mechanism for funding costly secondary and tertiary care for registered refugees is through the Exceptional Care Committees (ECC). The latter decides whether to finance or reject a refugee’s treatment based on criteria such as necessity of the treatment, financial need, disease prognosis and overall cost. The ECC finances a broad range of treatments [27].	Those requiring secondary or tertiary care need to be referred by a PHC center that works alongside the UNHCR except in the case of a life threatening circumstance [26]. UNHCR set guidelines for eligibility to subsidized secondary and tertiary care on life threatening conditions and likelihood of good prognosis. These include referrals for emergencies (obstetric, medical and surgical) and elective cases for complementary investigations and/or specific treatment [26]. If UNHCR’s criteria for hospital care are met, 75% of the treatment costs are covered, excluding cost of medicines, unless the patients meet UNHCR’s vulnerability criteria in which case 100% of costs are covered [26]. A private health care benefit management company assesses patients’ eligibility to access hospitals according to UNHCR guidelines. Cases that do not exactly fit eligibility criteria or where treatment costs over $1500 (with a cap of-$15,000) are submitted to the Exceptional Care Committee (ECC) that approves hospital admission [26, 39]. Refugees are covered only upon referral and for the first 24 h. After 24 h, refugees are responsible to seek approval for coverage. Life-saving conditions covered during the 24 h include myocardial infarction and respiratory distress. Cases not covered include long term sustaining tertiary care such as treatment/rehabilitation of complications of chronic degenerative diseases [42].	Referral is not required to access secondary and tertiary health care services provided by MOH [28]. Referral is required from state hospitals to access Ministry of Health affiliated university hospitals [29]*.* Those requiring referrals to health centres located in a different city than where the social insurance was issued, should seek referral at a state hospital where the person lives or at emergency services in case of an emergency [29]*.*

In Lebanon, registered refugees were able to access primary care at public primary health centers at a cost subsidized by UNHCR, but unregistered refugees were limited to health centers funded by private donors and charitable groups. Aside from life-threatening conditions such as myocardial infarction, gastrointestinal conditions that require surgical intervention, sepsis or septic shock, and respiratory distress, referral is needed for secondary and tertiary care, with specific eligibility criteria established by UNHCR. If these criteria were met, 75% of the treatment costs were covered, excluding cost of medicines, unless the patients met UNHCR’s vulnerability criteria in which case 100% of costs were covered [26]. Cases that do not fit eligibility criteria or where treatment costs over $1500, were submitted to the Exceptional Care Committee (ECC) for assessment of refugees’ eligibility to treatment financing based on criteria such as necessity of the treatment, financial need, disease prognosis and overall cost [27]. Any costs above $15,000 were to be made out of pocket.

In Jordan, access to primary and secondary care for Syrian refugees was free until November 2014. Since then, government policy required registered refugees with a Ministry of Interior card to pay a subsidized rate for care (similar to uninsured Jordanians); while refugees without such a card pay up to 60% more. Access to tertiary care required referral, and was not free of charge. UNHCR followed the same mechanism for funding secondary and tertiary care as in Lebanon.

In Turkey, AFAD provided free access to primary and secondary care for registered Syrian refugees, via existing community health centers and their referral networks for those living in host communities, and through field clinics and polyclinics for those in camps [28]. A 2013 field survey study revealed that over 90% of Syrian refugees living in camps used health services compared to approximately 60% of those living outside of camps [21]. No referral is required for secondary or tertiary care services provided by the Ministry of Health, unless referral is required for specialized care at a university hospital [28, 29]*.* A limited number of voluntary health centers run by non-governmental organizations with special permission from the Ministry of Health also provided primary health care for the refugees [28]. In 2015, Migrant Health Centers were established to address the overcrowding, linguistic barrier and the resentment towards Syrian refugees noted at some community health centers [30]. Unregistered refugees paid to access services similar to Turkish citizens without social insurance, except for emergency access to primary health care, which was provided for free [29].

### Access to NCD services for Syrian refugees

Access to NCD health services was reported in several studies in Jordan and Lebanon which assessed care-seeking practices in the host country, but there were no data available from Turkey. Of note, a 2014 Amnesty International report noted that some refugees returned to Syria to seek health services despite potential risks from the ongoing conflict [26]*.*

Although there were no differences noted in NCD care-seeking associated with UNHCR registration status, rates varied substantially across surveys. One survey reported that outside of camps in Jordan, of the 38% of refugees who sought primary care for chronic diseases, only half had received it [31], a lower proportion than those who sought care for acute conditions. In contrast, in a nationally representative study, also conducted outside camps in Jordan, 85% of those who sought care for five NCDs, received it, irrespective of the level of care [32], the highest number being for hypertension (78%). In two other surveys of Syrian refugees living outside camps in Jordan, and in host communities or informal tented settlements in Lebanon, 24 and 56% of household members with a chronic condition, respectively, were not able to access medicines or other health services [16, 17]. Whereas for refugees living within host communities or informal tented settlements in Lebanon, 83% of those who needed NCD care reported receiving it [15], the highest number being for diabetes mellitus (70%).

Refugees with arthritis reported less access in both countries (58% in Jordan and 54.4% in Lebanon) [15, 32]. It has been hypothesized that patients with arthritis are self-managing their condition with over-the-counter medications and do not find it necessary to seek health services. However, patients with arthritis may have reduced mobility and thus find it more difficult to travel to health facilities [33].

With regards to the type of health care services, in both Jordan and Lebanon, more than half of refugees sought care in primary health care centers. Although not specific to NCDs, public facilities in Jordan – whether primary health care centers or hospitals – were reported to be the preferred providers of care for 70% of non-camp refugees [31]. Fifty-four percent of Syrian refugees living outside camps in Jordan received care in the public sector, approximately 30% received care in the private sector and the rest received care in charity/non-governmental organizations [33]. In Lebanon, more than half of Syrian refugees received care in primary health care centers (58%), around a fifth at private clinics, and the rest at hospitals, community pharmacies or mobile medical units and home-based providers [15]*.* Hospitals were most often sought by refugees seeking care for cardiovascular diseases in Lebanon [15].

With regards to access to medications, approximately 85 and 75% of Syrian refugees with an NCD in Jordan and Lebanon, respectively, were taking their prescribed medications; and 26.5 and 31.6% reported stopping medication use or medication running out for longer than 2 weeks in the past year [13, 34]. A large percentage of refugees attending Caritas health centers in six Jordanian cities between November 2013 and June 2014 also reported not having enough medications (72%) [35]*.*

### Barriers to accessing NCD services

#### Financial barriers

Despite the availability of free or subsidized primary health services, the cost of treatment for NCDs is reported as the primary obstacle to accessing healthcare by Syrian refugees in Jordan and Lebanon [16, 17, 31, 32, 36–38]. Among Syrian refugees living outside camps and reporting hypertension, cardiovascular disease, diabetes mellitus, arthritis and/or chronic respiratory disease in Jordan, over half of those who did not seek care reported provider costs to be a barrier [32]. 45 and 79% of household members with a chronic illness living outside camps in Jordan, and in host communities or informal tented settlements in Lebanon, reported they could not afford user fees, respectively [16, 17]. A similar percent of Syrian refugees over 60 years old (*n* = 210) in Lebanon, identified cost as the main barrier to seeking consultation from a physician (79%) [25, 36].

A survey of Syrian refugees in Lebanon indicated that 70% of those seeking care for a chronic condition were paying out of pocket – including diagnostic and laboratory tests and excluding payments for medication – with the average consultation payment of 15USD [15]. Patients with cardiovascular disease, those accessing hospitals, and those using the private sector paid the most [15, 33]. In Jordan, 31.6% of refugees paid out-of-pocket for health services, with an average consultation payment of 18.8 USD [33].

Medication costs were a critical driver of overall out-of-pocket spending in both Jordan and Lebanon. In a 2013 survey of Syrian refugees living in three regions of Lebanon, more than half the respondents who were taking medicines for chronic diseases were paying directly for drugs [37]. Although nominally free or subsidized, medication stock-outs at public facilities required patients to purchase drugs, often at high cost [31, 34, 36–39]. In older refugees in Lebanon, 87% stated they had difficulty affording medications [25]. Many of the interviewees arranged for their medicines to be brought from Syria given their lower cost; as a short-term coping strategy [36].

Transportation was an additional financial barrier for Syrian refugees living outside camps in Jordan [13, 17, 32] and Lebanon whether they lived in host communities or informal tented settlements [16]; with 8 and 10% of non-camp Syrian refugees in the two countries respectively not accessing care as they could not afford transportation costs [16, 17].

These financial impediments had major consequences including adopting negative coping strategies, returning to Syria to seek treatment and most commonly, treatment interruptions [36, 40]. In Lebanon, 33–71% of those who needed treatment for NCDs suspended treatment due to costs [34, 41]; in Jordan, this is estimated at 59% [16]. Other strategies adopted to cope with healthcare expenditures [16, 26], included borrowing money, or relying on relatives or friends for payment [16], resulting in severe debts in some instances [26].

### Health systems barriers

#### Complex referral systems

Complex referral systems have limited refugees’ access to NCD care in Jordan and Lebanon [26, 31]. In Jordan, almost half of 103 non-camp Syrian refugees who reported difficulties in accessing care in a 2016 survey described complex referral as a main concern [31]. Procedures reportedly varied from center to center and overburdened staff were unable to provide the guidance necessary to navigate the referral process [31]. For example, the Irbid Jordan Health Aid Society (JHAS) clinic follows a protocol by which a physician approves referral to a public hospital where treatment is sponsored by UNHCR [31]. However, an alternative referral form is required when treatment is sought at other clinics supported by other non-governmental organizations that cater to patients with special needs [31]. In Lebanon, refugees reported lack of guidance regarding eligibility criteria and the absence of formal feedback/complaint mechanisms [26].

#### Lack of effective guidance on navigating the health system

Assessments also revealed limited levels of knowledge of available health services were found among refugees in the three host countries. A survey of older refugees in Lebanon revealed that in 2013, 12% lacked the knowledge about where to seek care and 7% did not know where to buy medications [25]. Ten percent of refugees living outside settlements in Lebanon did not know where to access medicine or other services, and only 24% were aware that a maximum of Lebanese Pounds 1000 (USD 0.67) was needed to refill prescription for chronic medication [16]. Interviews in 2014 conducted by Amnesty International with Syrian refugee patients revealed that eligibility criteria for attaining subsidized care remained unclear 3 years into the influx of refugees to Lebanon [26]. In Jordan, 15% of refugees living outside camps did not know where to access medications or other services in 2014 [17]. In a neighborhood in Istanbul, about half of refugee interviewees were not aware of availability of free access to health centers; with their main source of information being friends, neighbors and relatives (57.8%) followed by Turkish and Syrian doctors (13.3%) [20]*.*

#### Limited health facility capacity

The combination of high patient loads and limited working hours, may contribute to the reported long waiting times and delayed appointment dates; these in addition to complex procedures for services present substantive barriers to NCD management for Syrian refugees [16, 17, 31, 40, 42]. In addition, in Jordan and Lebanon, patient interviews indicated that lack of specialists is a major concern [31], particularly for complex cases that require specialized management [26, 37, 38]. Refugees also reported lack of trust in health providers [31, 32] and discrimination [16, 17, 31]. Although not limited to NCD care, a survey of non-camp refugees in Jordan found that 38% of respondents noting challenges in accessing care reported being rejected by a health facility or by its health personnel [31]. The study suggests that this may have been due to lack of understanding of the system on the part of refugees and/or health providers, or to health care workers intentionally not following procedures [31]*.*

Language constitutes a substantial barrier for Syrian refugees in Turkey, as very few Turkish health workers speak Arabic, and interpreters are scarce; one study reported inadequate translation of information relevant for access to care [43].

#### Health education for refugees with NCDs may also be suboptimal

In Jordan, a population-based health access assessment in non-camp settings found that 70% of refugees who self-reported at least one chronic condition (*n* = 51) received no health education while seeking care [38]. Similar findings were reported from Lebanon, where only 39% of refugees attending health facilities or mobile clinics of a local NGO reported receiving health education [42]. In Turkey, physicians who had been involved in one of the Syrian American Medical Society (SAMS) medical missions (*n* = 25) indicated spending around 20% of their time on health education, yet most of them doubted that this one intervention would have an impact on health behaviors, highlighting the need for adoption of a systematic way of promoting health education for patients [44].

## Discussion

To our knowledge, this review is the first to explore both the burden of NCDs amongst displaced Syrians living in Jordan, Lebanon and Turkey and the diverse health system responses. Given the pre-conflict estimates of NCD prevalence in Syria, it is not surprising that both community-based and facility-based surveys found a high prevalence of reported cardiovascular disease, hypertension, diabetes mellitus, chronic respiratory disease and other NCDs amongst Syrian refugees. However, the prevalence estimates varied greatly from study to study, likely due to diverse populations, age ranges, and survey methodologies with only two surveys using representative sampling [14, 34]. Most surveys relied on self- or proxy- reported diagnoses, raising the likelihood of underreporting of asymptomatic conditions, stigmatized diseases, and diagnoses requiring access to more complex diagnostic testing. In fact, the recently published WHO STEPS reports revealed a higher prevalence of measured hypertension among Syrian refugees in Lebanon (32.8%) and Turkey (25.6%) than the self- or proxy-reported prevalence included in this review, as well as a difference between measured and self-reported hypertension [14, 45]. A number of studies did not disaggregate NCD prevalence by age, likely underestimating the prevalence of these conditions among the adult population [18, 21, 22]*.*

Jordan, Lebanon and Turkey developed different approaches to these challenges (Table 2). Differences were influenced by the location of the refugees, whether in camps or outside camps, as well as by the presence/absence of refugee camps, and included varied entities providing the services. For example, in Turkey, the services are solely provided by the Turkish Government’s AFAD; whereas in Jordan and Lebanon, they involved UNHCR, Ministries of Health and NGOs. Another finding is that policies to support health services for refugees have changed over time. In Jordan, where access to primary and secondary care at public facilities was free of charge for UNHCR registered refugees until late 2014, thereafter Syrian refugees were required to pay the same fee as uninsured Jordanians. More recently, in February 2018, the Jordanian government decreed that Syrian refugees have to pay the rates that non-citizens are required to pay when seeking medical care minus 20%, rather than the rates for uninsured Jordanians, which is likely to further raise costs and discourage health-seeking.

Our review found that access to NCD services varied both within and between countries, with no data available from Turkey; and that out of pocket costs were a significant barrier in Jordan and Lebanon. As funding for the Syrian crisis falls short of needs by almost half [2], assistance to refugees will likely decrease, and cost will increasingly become a barrier for access to care. Furthermore, Jordan’s 2018 policy change is likely to markedly affect access to secondary and tertiary care. For example, the World Bank reported a 60% decrease in health service utilization by refugees in Jordan within 2 years of implementation of the co-payment policy [46]. In addition, an Amnesty International reported a 27% increase in the number of patients seeking treatment at the Jordan Health Aid Society, an organization which assists vulnerable Syrians in getting access to care [47]. In addition to financial and geographic barriers, complex referral systems and bureaucracies pose challenges to refugees attempting to navigate health systems in Lebanon and Jordan, while the language barrier was a substantial challenge for Syrian refugees in Turkey seeking health services.

Turkey, Jordan and Lebanon have adopted integrated approaches to planning, delivery and financing of NCD services by embedding refugee health care within the national health systems. However, gaps remain, highlighting the advantage of universal health coverage to reduce inequalities faced by refugee populations, and vulnerable host populations. The World Bank has recently approved two emergency health projects in Jordan and Lebanon; the Jordan Emergency Health Project and the Lebanon Health Resilience Project in which innovative financing mechanisms will be implemented to strengthen the capacity of national health systems to provide quality services to national vulnerable populations and Syrian refugees [48, 49].

Access to health insurance may lead as well to improved access to secondary and tertiary NCD services; as in the example of Iran where health insurance for Afghan refugees in 2011 led to better access and reduced risk of having to pay for hospitalization of refugees [50]*.* This strategy is more likely to succeed in countries where refugees are legally permitted to work and, thus, are able to afford insurance premiums or co-payments while UNHCR focuses on supporting vulnerable persons [50].

Innovations may also assist in expanding access to services and enhancing the quality of the services. Given the high penetration of smart phones among Syrian refugees [51, 52], digital solutions may improve navigation and supporting continuity of care. In one project, the one project, the use of an mHealth application at primary healthcare centers in Lebanon had a positive effect on follow up appointments and patient/provider interactions among a cohort of Syrian refugees accessing treatment for hypertension and diabetes mellitus [53]. The use of community radio health programs have also been suggested as a means of providing Syrian women refugees with reproductive and antenatal health education, as well as a medium in which they can express their health concerns and ask a healthcare provider questions without having to go to the primary healthcare clinics [54].

This is the first review of the published and grey literature on the burden of NCDs and access to NCD services among Syrian refugees in neighboring host countries. The study strengths include the comprehensive approach to identifying resources from the published, grey and not-published literature, including direct outreach to stakeholders for unpublished data. In addition, the trilingual study staff were able to review English, Arabic and Turkish resources, mitigating previously noted language barriers to reviews on this subject. However, this review has several limitations. Although the authors cross-checked their results from Google and key agency websites, it is possible that they may have missed some grey literature resources. Other weaknesses include the fact that no standardized tool was used to assess the quality of included studies/reports, the limited information on access to NCD services in Turkey, the lack of clear distinctions between care-seeking, coverage and access to NCD services in some of the surveys in Jordan and Lebanon [55], and that most surveys collected self- or proxy reported information on NCDs rather than diagnosed conditions which may have underestimated prevalence of various NCDs. Much of the retrieved evidence is descriptive and has not addressed key aspects in NCD management such as models of care, continuity or quality of care. Thus, future research should consider filling these knowledge gaps and in-depth analyses are required to better identify the role of such factors in limiting NCD care.

## Conclusions

Over the past 7 years, Turkey, Jordan and Lebanon have made extraordinary efforts to respond to the Syrian refugee crisis, particularly in terms of provision of health services. These efforts are affected both by the impact of millions of displaced people on host health systems, and by the complex health needs of refugees from a middle-income country with a high prevalence of NCDs. Provision of high-quality continuity NCD services at scale has been challenging and may become more so given ongoing and anticipated funding shortfalls. Innovative service delivery models, ongoing advocacy, rigorous evaluation and implementation science methods and the empowerment of displaced persons and health workers will be necessary to create responsive and sustainable solutions to the threat of NCDs.

## Additional file

Additional file 1: PUBMED Search Strategy. (DOC 25 kb)

## Abbreviations

AFAD: Disaster and Emergency Management Presidency
ECC: Exceptional Care Committee
JHAS: Jordan Health Aid Society
MSF: Médecins Sans Frontières
NCD: Non-communicable disease
NGO: Non-governmental organization
STEPS: STEPwise approach to Surveillance
UNHCR: United Nations High Commissioner for Refugees

## Footnotes

1 Ranging between surveys that selected representative samples of a camp or region (but not at country level) and those that used a convenience sampling or snowball method
