# Supplementary material for: Host country responses to non-communicable diseases amongst Syrian refugees: a review
Source: Confl Health. 2019 Mar 22;13:8. doi: 10.1186/s13031-019-0192-2 (PMC6431037; doi:10.1186/s13031-019-0192-2)
Supplement: Supplementary file 1 — PUBMED Search Strategy. (DOC 25 kb) [file 13031_2019_192_MOESM1_ESM.doc]

**Additional file 1: PUBMED Search Strategy**

"neoplasms" OR neoplasm* OR "osteoporosis" OR osteoporosis OR "osteoarthritis" OR "arthritis" OR rheumatoid arthritis OR "copd" OR COPD OR "asthma" OR asthma OR "cancer" OR cancer* OR "renal disease" OR renal disease* OR "kidney disease" OR kidney disease* OR "hyperglycaemia" OR "insulin resistance" OR insulin resistan* OR "hyperinsulinemia" or hyperinsulinem* OR "diabetes mellitus/type 2" OR diabetes mellitus* OR "embolus" OR embolus OR "stroke" OR stroke OR "hypertriglyceridemia" OR hyper triglyceridemia OR hypertriglyceridemia OR "high triglyceride" OR high triglyceride* OR "high cholesterol" OR high cholesterol OR "hypercholesterolemia" OR hypercholesterolemia OR "dyslipidemia" OR dyslipidemia* OR "hyperlipidemia" OR hyperlipidemia* OR "diastolic pressure" OR diastolic pressure OR "systolic pressure" OR systolic pressure OR "blood pressure" OR blood pressure OR "hypertension" OR hypertension OR "cardiometabolic syndrome" OR cardiometabolic syndrome* OR "metabolic syndrome x"OR "syndrome x" OR syndrome X OR "metabolic syndrome" OR MetS OR metabolic syndrome OR "coronary heart disease" OR coronary heart disease* OR "cvd" OR CVD* OR "chd" OR CHD* OR "ncd" OR NCD* OR "cardiovascular event" OR "cardiovascular disease" OR cardiovascular AND disease* OR event* OR "coronary disease" OR coronary disease* OR "nutrition related chronic diseases" OR nutrition related chronic disease* OR "non communicable disease" OR non communicable disease* OR "obesity" or obes*) OR "smoking"

OR

"health care services" OR healthcare service* OR "health service" OR "health care" OR health service* OR healthcare OR "health seeking" OR health seek* OR "health utilisation" OR health utilization OR health utilization OR "health work force" OR health workforce OR "human resource" OR human resources* OR "field hospital" OR field hospital* OR field clinic* OR mobile clinic* OR "primary health care"

AND

(("syria"[Title/Abstract] OR Syria*[Title/Abstract])) NOT (hamster* OR syriaca)
